# Explainable machine learning model for predicting hearing recovery in unilateral sudden sensorineural hearing loss

**DOI:** 10.1016/j.bjorl.2025.101730

**Published:** 2025-12-17

**Authors:** Jueting Wu, Ruru Chen, Yaxuan Liu, Feng Zhao, Huiying Chen, Xiaoyu Lin, Jiping Su

**Affiliations:** a The First Affiliated Hospital of Guangxi Medical University, Department of Otolaryngology-Head and Neck Surgery, Nanning, Guangxi, China; b Department of Otolaryngology-Head and Neck Surgery, Lishui Hospital of Wenzhou Medical University, The First Affiliated Hospital of Lishui University, Lishui People's Hospital, Lishui, Zhejiang 323000, China; c The Second Affiliated Hospital and Yuying Children’s Hospital of Wenzhou Medical University, Department of Otolaryngology-Head and Neck Surgery, Wenzhou, Zhejiang, China

**Keywords:** Machine learning, Sudden sensorineural hearing loss, Serum albumin, Mean corpuscular volume, Shapley additive explanations

## Abstract

**Objective:**

Sudden Sensorineural Hearing Loss (SSNHL) is routinely encountered in otolaryngology clinics. The prognosis of SSNHL varies dramatically and depends on multiple influence factors. This study aimed to develop an explainable Machine Learning (ML) model with easily accessible features to predict the prognosis of SSNHL.

**Methods:**

This bi-center retrospective study included 534 patients with SSNHL. We randomly split the data into training and validation sets. Univariate analysis and Least Absolute Shrinkage and Selection Operator (LASSO) regression were employed to select predominant features, including demographic, disease-specific characteristics, and laboratory items. We evaluated the performance of five ML models constructed with six crucial variables using the Area Under the receiver operating characteristic Curve (AUC), accuracy, specificity, sensitivity, and F1 scores. These models were further calibrated by calibration curve and Brier score. Clinical utility was evaluated by Decision Curve Analysis (DCA). The Shapley Additive Explanations (SHAP) method was applied to interpret feature contribution and explain the ML models.

**Results:**

The Random Forest (RF) model reached the highest AUC of 0.998. Its accuracy, sensitivity, specificity, and F1 score were 0.981, 0.963, 0.989, and 0.967, respectively. DCA curve analysis revealed a comparable net benefit of the models. The SHAP method revealed that the primary features were degree of hearing loss, audiogram type, age, Mean Corpuscular Volume (MCV), onset to treatment, and serum Albumin (ALB) accordingly.

**Conclusions:**

The explainable ML model was superb in predicting hearing outcome and providing information on feature contributions. Clinicians can better understand the contributors to hearing recovery and guide aural rehabilitation using the SHAP method.

**Level of evidence:**

Level 3.

## Highlights

• Explainable ML performed well in predicting hearing recovery in SSNHL patients.
• The RF model performed best among the ML models
• The most primary feature was degree of hearing loss.
• MCV was found to be an pivotal factor for the first time.
• The SHAP method help clinicians to better understand the features in ML models.

## Introduction

Sudden Sensorineural Hearing Loss (SSNHL) is commonly encountered in otological emergencies and requires immediate medical attention. Despite extensive research, hearing recovery following SSNHL can occur, be partial, or complete.^1^ Therefore, early identification of principal predictive factors are conducive to forecasting recovery, monitoring treatment, and guide aural rehabilitation.

Recently, Machine Learning (ML) has demonstrated promising results in predicting the prognosis of SSNHL due to its capacity for handling complex interactions and non-linear relationships.^2^, ^3^, ^4^, ^5^, ^6^ However, this method is challenging to interpret. Understanding the decision-making processes and output generated by ML algorithms comprehensively will increase the acceptance of confidence in prediction models. Therefore, we established ML models based on Shapley Additive Explanations (SHAP). This visualization method is capable of assessing the importance of each variable’s contribution independently even if the variables are intricately related. Previous studies have mainly focused on developing machine learning models by using a large number of variables in their algorithms. Optimistic results and overfitting may occur under such circumstances, limiting the generalizability of the models in the meantime. The objective of this study was to develop an explainable ML model with fewer easily accessible variables to predict hearing recovery of SSNHL.

## Methods

### Patient selection

**Fig. 1:**
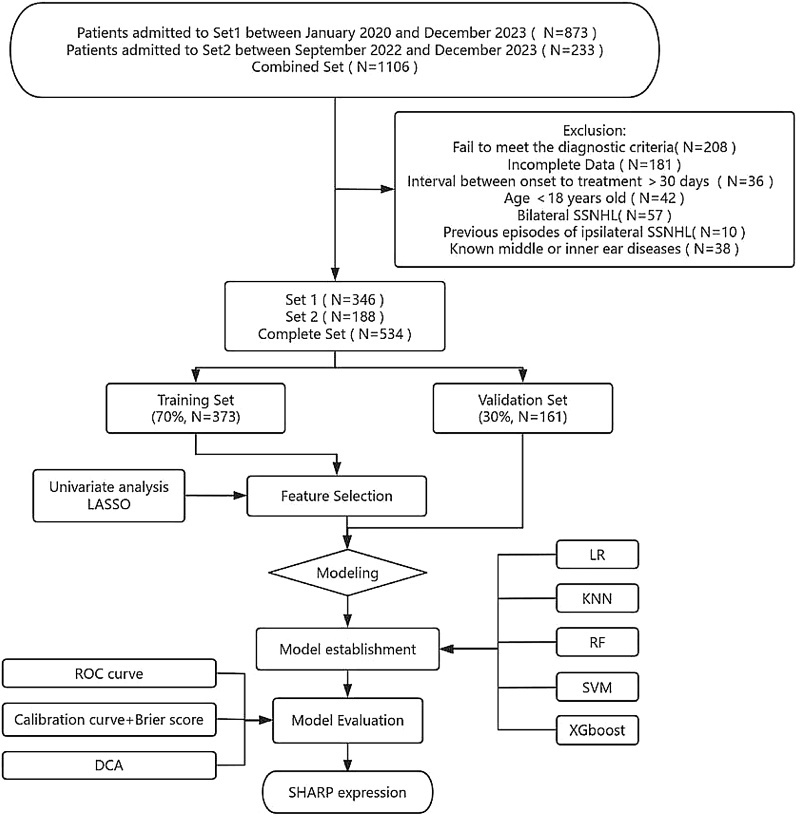
Flow chart of the study design. LASSO, Least Absolute Shrinkage and Selection Operator; LR, Logistic Regression; KNN, K-Nearest Neighbor; RF, Random Forest; SVM, Support Vector Machine; XGBoost, Extreme Gradient Boosting; ROC, Receiver Operating Characteristic; DCA, Decision Curve Analysis; SHAP, Shape Additive Explanation.

The data sets were obtained from Set1 between January 2020 and December 2023, and Set2 between September 2022 and December 2023. We reviewed patients with unilateral SSNHL under the diagnostic criteria of sudden hearing loss (≥30 dB) for at least three consecutive frequencies within 72 h.^1^ Patients with PTA data at the start and a month after treatment were included in the analysis. Patients with any of the following characteristics were excluded: (1) Age < 18-years, (2) Interval between onset to treatment >30-days, (3) Previous episodes of ipsilateral SSNHL, and (4) Known middle or inner ear diseases. The final analysis included 534 patients out of 1106 screened patients from these two institutions (Fig. 1).

This retrospective study was performed in line with the principles of the Declaration of Helsinki, and approved by the Ethics Committee. The requirement for informed consent was waived because the study met the criteria for minimal risk to the study participants.

### Audiometric assessment

The grades of hearing loss was defined as follow^7^: normal hearing, mild hearing loss, moderate hearing loss, moderately severe hearing loss, severe hearing loss, profound hearing loss, and total hearing loss.

Audiogram shapes were identified based on the pattern of hearing loss: ascending, flat, profound, and descending.^8^ The average threshold of 250–500 kHz showed 20 dB higher or lower than the average threshold of 4–8 kHz was classified as “Ascending or descending”, “flat” referred to the difference in hearing threshold less than 20 dB at any frequency, a flat audiogram that had a hearing threshold above 90 dB was labeled as “profound”.

Siegel’s criteria was used to define hearing recovery using the average values of 500 Hz, 1 kHz, 2 kHz, 4 kHz^9^: (1) Complete recovery meant final hearing level <25 dB; (2) Partial recovery included hearing gain >15 dB and final hearing level between 25 and 45 dB; (3) Slight recovery included hearing gain >15 dB and final hearing >45 dB; and (4) No improvement included hearing gain <15 dB, or final hearing threshold >75 dB. We defined hearing recovery as complete recovery and partial recovery, while no recovery included slight recovery and no improvement.

### Treatment protocols

Routine blood tests were performed upon admission. All patients were treated with systemic corticosteroids and hemodilution agents following the Chinese guidelines for the diagnosis and treatment of sudden deafness.^10^ Afterward, the patients continued to take oral medication and were monitored in the outpatient clinic.

### Machine learning models construction

A total of 80 variables including demographic features, disease-specific features and laboratory variables were enrolled. Features with more than 20% missing values were excluded, and others were handled by Multivariate Imputation Chained Equations (MICE) imputation strategy. Significant variables (p < 0.1) selected by univariate analysis between recovery and non-recovery group were applied for further analysis. We randomly split the data into two parts for training (70%) and validation (30%). Predictors were filtered in the training set using the Least Absolute Shrinkage and Selection Operator (LASSO) analysis. Using the six pivotal LASSO selected variables, five machine learning models: Random Forest (RF), Logistic Regression (LR), Support Vector Machine (SVM), K-Nearest Neighbor (K-NN), and extreme Gradient Boosting (XGBoost) were created and evaluated. Five-fold Cross-Validation (CV) was used to train and verify the models for 10 repetitions.

The performance of the ML models was assessed using the Receiver Operating Characteristic (ROC) curve, which included the AUC, accuracy, specificity, sensitivity, and F1 scores. The calibration curve and the Brier score further validated their predictive power. The Decision Curve Analysis (DCA) curve was created to evaluate the clinical applicability of the models. To explain the RF model, we used SHAP values to interpret the effects of features on prediction and interpret each predictive factor at the patient level.

### Statistical analysis

Mean Standard Deviation (SD) or median (Q1, Q3) were used to present continuous variables, and percentages were displayed for the categorized variables. The non-recovery and recovery groups were identified by using *t*-tests, Mann–Whitney *U*-tests, Chi-Square tests, and Fisher's exact test. The correlations among variables were analyzed using Spearman correlation. Delong's method was used to compare AUC between models. The best critical value (cutoff value) was determined by using the Youden index (sensitivity + specificity-1). All analyses were conducted using *R* software (version 4.2.3) and IBM SPSS 25.0 (IBM, Armonk, NY, USA); p < 0.05 were used to denote statistical significance.

## Results

### Patients’ characteristics

We initially included 873 participants from Set1 and 233 participants from Set2. After exclusion, a total of 534 participants were analyzed (Fig. 1). Out of the participants, 159 (29.8%) experienced recovery, while 375 (70.2%) did not. The entire cohort had a median age of 50 (39, 60) years. Hearing loss recovery was more likely to occur in patients with lower degree of hearing loss, younger age, and shorter hospital stay. Ascending and flat audiogram shapes present a higher possibility of recovery. Furthermore, patients in the recovery group had a significantly greater proportion of aural fullness and a lower percentage of vertigo. The non-recovery group is more prone to developing hypertension. Patients in the non-recovery group had significantly decreased serum Albumin (ALB) levels, Monocyte percentage (MONO%), Red Blood Cell Count (RBC), and Albumin/Globulin ratio (A/G), whereas the Mean Corpusular Hemoglobin (MCH), Mean Corpusular Volume (MCV) level, Blood Urea Nitrogen (BUN) value, Apolipoprotein B (ApoB) value, serum Sodium (Na), Thrombin Time (TT), and BUN and Creatinine ratio (BUN/Cr) were elevated remarkably. The distribution of sex affected side, addiction, comorbidities, and BMI did not significantly differ between the two groups.

The training set included 373 patients and the validation set had 161, with a ratio of 7:3. There was no statistical difference between all predictive variables in both sets. Table 1 summarizes the patient characteristics.

**Table 1:** Patient characteristics between groups (non-recovery group and recovery group) and sets (training set and validation set) (n = 534).

	Overall (n = 534)	Non-Recovery Group (n = 375)	Recovery Group (n = 159)	p-value	Training Set (n = 373)	Validation Set (n = 161)	p-value
**Characteristics**							
Outcome(recovery), n (%)					107 (29)	52 (32)	0.463
Demographic data							
Age, Median (Q1, Q3)	50 (39, 60)	53 (43, 61)	42 (31, 51)	<0.001	49 (38, 60)	51 (40, 61)	0.391
Sex(male), n (%)	248 (46)	171 (46)	77 (48)	0.614	172 (46)	76 (47)	0.89
Current Smoker, n (%)	93 (17)	69 (18)	24 (15)	0.426	67 (18)	26 (16)	0.702
Current drinker, n (%)	61 (11)	49 (13)	12 (8)	0.092	40 (11)	21 (13)	0.532
Hypertension, n (%)	115 (22)	90 (24)	25 (16)	0.044	77 (21)	38 (24)	0.517
Diabetes, n (%)	62 (12)	48 (13)	14 (9)	0.242	45 (12)	17 (11)	0.725
Cardiac disease, n (%)	11 (2)	9 (2)	2 (1)	0.519	7 (2)	4 (2)	0.741
Cerebrovascular accident, n (%)	9 (2)	9 (2)	0 (0)	0.064	5 (1)	4 (2)	0.463
Disease-specific data							
Affected side(left), n (%)	290 (54)	201 (54)	89 (56)	0.683	208 (56)	82 (51)	0.35
Degree of hearing loss				<0.001			0.481
Normal	2 (0)	0 (0)	2 (1)		1 (0)	1 (1)	
Mild	23 (4)	5 (1)	18 (11)		18 (5)	5 (3)	
Moderate	54 (10)	31 (8)	23 (14)		36 (10)	18 (11)	
Moderately severe	91 (17)	45 (12)	46 (29)		66 (18)	25 (16)	
Severe	105 (20)	63 (17)	42 (26)		66 (18)	39 (24)	
Profound	94 (18)	76 (20)	18 (11)		70 (19)	24 (15)	
Total	165 (31)	155 (41)	10 (6)		116 (31)	49 (30)	
Audiogram shape, n (%)				<0.001			0.266
Ascending	73 (14)	25 (7)	48 (30)		45 (12)	28 (17)	
flat	180 (34)	112 (30)	68 (43)		125 (34)	55 (34)	
descending	106 (20)	73 (19)	33 (21)		80 (21)	26 (16)	
profound	175 (33)	165 (44)	10 (6)		123 (33)	52 (32)	
Tinnitus, n (%)	484 (91)	346 (92)	138 (87)	0.068	339 (91)	145 (90)	0.891
Aural fullness, n (%)	130 (24)	73 (19)	57 (36)	<0.001	91 (24)	39 (24)	1
Vertigo, n (%)	167 (31)	140 (37)	27 (17)	<0.001	123 (33)	44 (27)	0.234
Onset to treatment, Median (Q1, Q3)	4 (2, 7)	4 (2, 7)	4 (2, 7)	0.061	4 (2, 7)	4 (2, 7)	0.814
LOS, Median (Q1, Q3)	7 (6, 9)	7 (6, 9)	7 (5, 8.5)	0.012	7 (6, 9)	7 (6, 9)	0.395
BMI, Median (Q1, Q3)	23.68(21.78, 25.91)	23.69 (21.8, 25.86)	23.67 (21.63, 25.93)	0.808	23.67 (21.76, 25.87)	23.81 (21.97, 25.93)	0.751
Laboratory data							
NLR, Median (Q1, Q3)	3.04 (2.1, 5.2)	3.15 (2.11, 5.35)	2.97 (1.99, 4.88)	0.379	3.2 (2.17, 5.35)	2.81 (1.97, 5.01)	0.293
CRP, Median (Q1, Q3)	1.02 (0.3, 2.57)	0.98 (0.3, 2.41)	1.1 (0.3, 2.6)	0.667	1.04 (0.3, 2.58)	1 (0.3, 2.15)	0.618
ALB, Mean ± SD	43.66 ± 3.5	43.28 ± 3.37	44.55 ± 3.65	<0.001	43.56 ± 3.46	43.88 ± 3.59	0.344
A/G, Mean ± SD	1.65 ± 0.27	1.63 ± 0.27	1.69 ± 0.24	0.009	1.64 ± 0.26	1.67 ± 0.27	0.26
CRP/ALB, Median (Q1, Q3)	0.02 (0.01, 0.06)	0.02 (0.01, 0.05)	0.03 (0.01, 0.06)	0.371	0.03 (0.01, 0.06)	0.02 (0.01, 0.06)	0.212
FIB/ALB, Median (Q1, Q3)	0.06 (0.05, 0.07)	0.06 (0.05, 0.07)	0.06 (0.05, 0.07)	0.405	0.06 (0.05, 0.07)	0.06 (0.06, 0.07)	0.531
MONO%, Median (Q1, Q3)	0.06 (0.04, 0.07)	0.05 (0.04, 0.07)	0.06 (0.05, 0.07)	0.034	0.06 (0.04, 0.07)	0.05 (0.04, 0.07)	0.571
MONO, Median (Q1, Q3)	0.45 (0.3, 0.63)	0.44 (0.3, 0.61)	0.48 (0.34, 0.67)	0.087	0.46 (0.3, 0.64)	0.42 (0.3, 0.55)	0.083
RBC, Median (Q1, Q3)	4.57 (4.19, 4.9)	4.52 (4.19, 4.86)	4.67 (4.26, 4.97)	0.006	4.59 (4.21, 4.93)	4.54 (4.17, 4.87)	0.405
MCV, Median (Q1, Q3)	91.4 (88.32, 93.9)	91.7 (89, 94.2)	90.4 (86.75, 93)	<0.001	91.2 (88.2, 93.9)	91.7 (88.7, 93.9)	0.498
MCH, Median (Q1, Q3)	30.3 (29.4, 31.3)	30.4 (29.5, 31.4)	30.1 (29.1, 31.1)	0.032	30.2 (29.4, 31.2)	30.5 (29.6, 31.4)	0.237
TP, Mean ± SD	70.66 ± 5.34	70.41 ± 5.36	71.25 ± 5.27	0.092	70.65 ± 5.37	70.69 ± 5.31	0.936
BUN, Median (Q1, Q3)	5.4 (4.58, 6.4)	5.5 (4.6, 6.6)	5.22 (4.35, 6)	0.02	5.32 (4.5, 6.37)	5.6 (4.79, 6.6)	0.089
UA, Median (Q1, Q3)	297 (242, 376.75)	295 (242, 361.5)	313 (242, 401.5)	0.068	296 (240, 377)	307 (247, 375)	0.399
BUN/Cr, Median (Q1, Q3)	0.09 (0.07, 0.1)	0.09 (0.07, 0.11)	0.08 (0.07, 0.1)	0.017	0.09 (0.07, 0.1)	0.09 (0.07, 0.1)	0.508
ApoB, Median (Q1, Q3)	1 (0.73, 1.32)	1.04 (0.78, 1.34)	0.86 (0.67, 1.24)	0.004	0.99 (0.71, 1.32)	1.03 (0.77, 1.39)	0.179
Na, Median (Q1, Q3)	140.8 (139.2, 142)	141 (139.4, 142.4)	140.5 (138.95,141.45)	0.005	140.7(139.1, 142.1)	140.9 (139.2, 141.9)	0.541
Cl, Mean ± SD	105.23 ± 2.73	105.37 ± 2.79	104.89 ± 2.56	0.057	105.31 ± 2.71	105.03 ± 2.78	0.281
TT, Median (Q1, Q3)	17 (16.5, 17.6)	17 (16.5, 17.6)	16.8 (16.3, 17.4)	0.004	16.9 (16.5, 17.6)	17 (16.5, 17.6)	0.928

LOS, Length of Hospital Stay; BMI, Body-Mass Index; NLR, Neutrophil to Lymphocyte Ratio; CRP:C-Reactive Protein; ALB, Serum Albumin; A/G, Albumin to Globulin ratio; CRP/ALB, C-Reactive Protein/Albumin ratio; FIB/ALB, Fibrinogen to Albumin Ratio; MONO%, Monocyte Percentage; MONO, Monocyte count; RBC, Red Blood Cell Count; MCV, Mean Corpusular Volume; MCH, Mean Corpusular Hemoglobin; TP, Total Protein; BUN, Blood Urea Nitrogen; UA, Uric Acid; BUN/Cr, BUN to Creatinine ratio; ApoB, Apolipoprotein; Na, Serum Sodium; Cl, Chlorine; TT, Thrombin Time. Continuous data are expressed as mean ± SD or Median (Q1, Q3); Nominal data are expressed as n (%).

### Feature selection

**Fig. 2:**
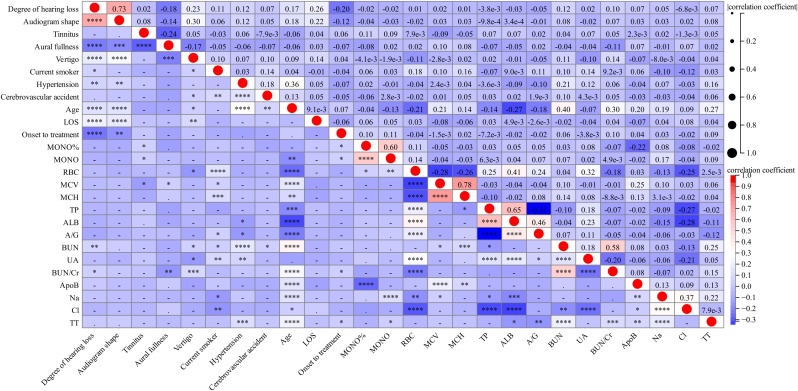
Correlation among 26 selected variables selected by univariate analysis. Red indicates positive correlation; blue indicates negative correlation. “****”, p < 0.0001; “***”, p < 0.001; “**”, p < 0.01; “*”, p < 0.05;“-”, no significance.

**Fig. 3:**
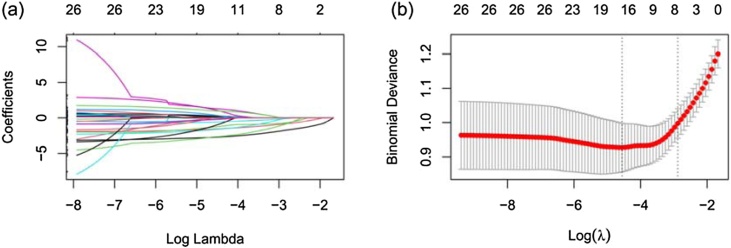
Feature selection using the LASSO regression.

A significant correlation was observed among 26 features selected by univariate analysis (Fig. 2). Therefore, we employed LASSO regression to reduce the dimension and avoid multicollinearity and overfitting among these variables.^11^ Subsequently, sixteen features were selected (Fig. 3a‒b). We chose the top six characteristic variables for model construction, including the degree of hearing loss, audiogram shape, age, duration between onset to treatment, ALB, and MCV level.

### Model evaluation and interpretation

**Fig. 4:**
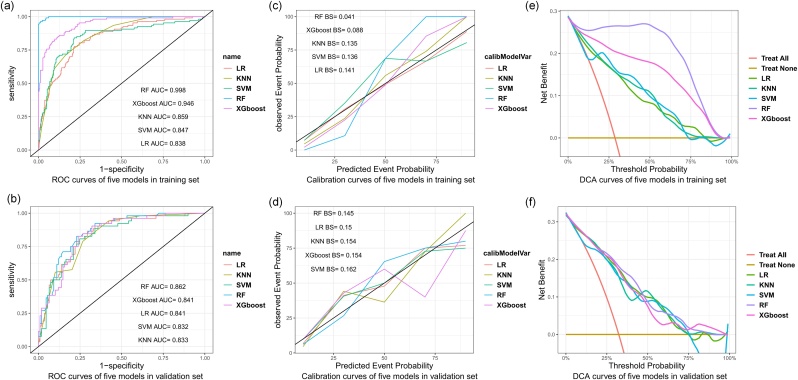
Evaluation and validation of model performance. Receiver Operating Characteristic Curves (ROC) of five machine learning models in the training and validation sets (a‒b). The calibration curve and Brier score of five machine learning models in the training and validation sets (c‒d). The decision curve analysis of five machine learning models in the training and validation sets (e‒f).

Fig. 4a and Table 2 showed that the RF model had the best performance in the training set with an AUC of 0.998. The accuracy, sensitivity, specificity, and F1 score was 0.981, 0.963, 0.989, and 0.967, respectively. The LR model performed poorer than others with an AUC of 0.838, accuracy of 0.788, sensitivity of 0.776, specificity of 0.786, and F1 score of 0.672. The AUC of the RF model differed significantly from the other models (p < 0.0001). Five models in the validation set had AUCs above 0.8, and the RF model also reached a higher AUC value of 0.862 (Fig. 4b). Table 2 summarizes the results of different algorithms’ performance. Fig. 4 c–d showed that the predicted probability of the models was close to the observed probability, as the calibration curve for each algorithm was close to the 45 ° line. Meanwhile, the Brier score was lowest for the RF model (Brier score = 0.041) compared to the other models. DCA analysis revealed that the RF model had significant net benefits for threshold probabilities at various time points(Fig. 4e‒f). All models showed a comparable net benefit within the threshold probability of 0.75, suggesting the potential clinical benefit of the models.

**Table 2:** Evaluation of model performance in the training set and validation set.

Models	AUC (95% CI)	Accuracy (95% CI)	Sensitivity (95% CI)	Specificity (95% CI)	F1 score
Training Set					
RF	0.998 (0.996‒0.999)	0.981 (0.962‒0.992)	0.963 (0.927‒0.999)	0.989 (0.976‒1)	0.967
XGBoost	0.946 (0.919‒0.969)	0.869 (0.830‒0.901)	0.860 (0.794‒0.926)	0.876 (0.836‒0.916)	0.793
K-NN	0.859 (0.819‒0.896)	0.802 (0.758‒0.841)	0.710 (0.624‒0.796)	0.838 (0.794‒0.883)	0.673
SVM	0.847 (0.796‒0.894)	0.802 (0.758‒0.841)	0.841 (0.772‒0.910)	0.786 (0.736‒0.835)	0.708
LR	0.838 (0.790‒0.881)	0.788 (0.737‒0.824)	0.776 (0.697‒0.855)	0.786 (0.736‒0.835)	0.672
Validation Set					
RF	0.862 (0.796‒0.916)	0.789 (0.718‒0.849)	0.827 (0.724‒0.930)	0.771 (0.692‒0.850)	0.716
XGBoost	0.841 (0.782‒0.899)	0.783 (0.711‒0.844)	0.827 (0.724‒0.930)	0.761 (0.681‒0.841)	0.711
K-NN	0.833 (0.765‒0.891)	0.752 (0.677‒0.816)	0.788 (0.677‒0.899)	0.734 (0.651‒0.817)	0.672
SVM	0.832 (0.761‒0.893)	0.770 (0.697‒0.833)	0.808 (0.701‒0.915)	0.752 (0.671‒0.833)	0.695
LR	0.841 (0.776‒0.900)	0.758 (0.684‒0.822)	0.808 (0.701‒0.915)	0.752 (0.671‒0.833)	0.697
Comparisons of AUC among models (p-value)					
	**RF vs. XGBoost**	**RF vs. K‒NN**	**RF vs. SVM**	**RF vs. LR**	**SVM vs.LR**
Training Set	<0.0001	<0.0001	<0.0001	<0.0001	0.607
Validation Set	0.061	0.147	0.126	0.142	0.601

**Fig. 5:**
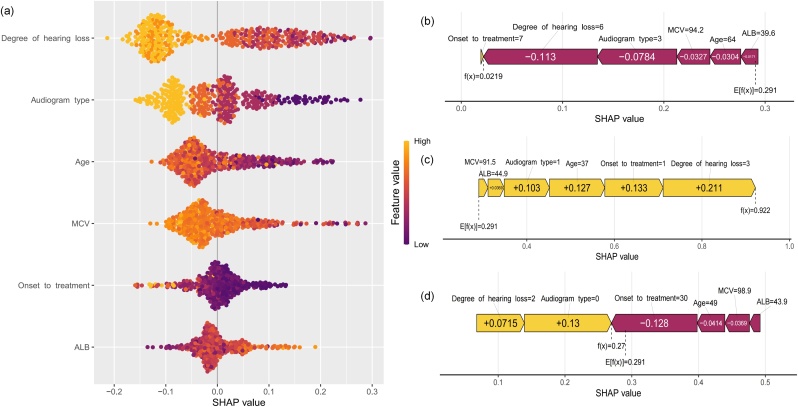
Shap explanation at patient level. The summary plot of the RF model (a). Each point on the summary plot is a Shapley value for a feature and an instance. The position on the y-axis is determined by the feature and on the x-axis by the Shapley value. The color from yellow to purple represents the value of the feature from high to low. The SHAP force plot for predicting individual hearing recovery (b‒d). Correctly predict non-recovery (b); correctly predict recovery (c); a detailed explanation of a random patient (d). Grade of hearing loss from 0 to 6 represent normal, mild, moderate, moderately severe, severe, profound and total hearing loss accordingly. Audiogram shape from 0 to 3 represent ascending, flat, descending and profound shape accordingly.

The SHAP summary plot displays how features contribute to the RF model (Fig. 5a). The most important feature was the degree of hearing loss, followed by audiogram type, age, MCV, onset to treatment duration, and ALB. According to the prediction model, a lower likelihood of hearing recovery is associated with a higher degree of hearing loss, MCV levels and lower ALB levels. A ascending audiogram shape is typically associated with better hearing recovery, followed by the flat type. The descending and profound audiograms tend to correlate with the maintenance of hearing loss, especially the profound shape, which has the poorest prognosis. Additionally, older age was a negative predictor of hearing outcomes.

### Sharp explanation of individual patients

To assess the impact of features on individual patients, we applied the SHAP method to explain the individual predictions from several random cases, including hearing recovery and non-recovery. Fig. 5b shows the SHAP force plot to accurately predict non-recovery; the prediction model was supported by the Shapley value of complete hearing loss, profound audiogram shape, greater MCV level, older age, lower ALB level, shorter onset to treatment duration. With a Shapley value of 0.0219 compared to the baseline value of 0.291, the case was correctly predicted. Fig. 5c shows a case of predicting hearing recovery; the predictive model was supported by the Shapley value of moderately severe hearing loss, shorter onset to treatment duration, younger age, flat audiogram shape, greater ALB level, and lower MCV level, with a Shapley value of 0.922.

Fig. 5d presents a 49-year-old case who sought medical advice 30 days after experiencing hearing impairment. The initial hearing assessment showed moderate hearing loss and an ascending audiogram shape. An elevated MCV and decreased ALB were detected. Our predicted Shapley value was 0.27, which was below the average value of 0.291 and led to a negative outcome. The results also suggested that an ascending audiogram shape and a prolonged onset to treatment duration were the main contributors.

## Discussion

Early identification of vital prognostic factors is crucial for determining the appropriate treatment plan and maximizing the chances of hearing recovery. This study focused on exploring desired predictive features and shedding light on model interpretation for every individual patient. By using SHAP method, we were able to identify the pivotal predictors of hearing recovery. The SHAP results showed that the degree of hearing loss, audiogram shape, duration between onset to treatment, age, MCV, and ALB levels, which can be expediently assessed in clinical practice, are crucial features to predict hearing recovery. This insight ensured clarity for clinicians and boost acceptance on ML models.

The present findings suggested that the degree of hearing loss was considered the most important predictor. The hearing recovery rate differed greatly among different hearing levels in our study. 18 out of 23 (78.3%) patients with mild hearing loss achieved hearing recovery, while only 10 out of 165 (6.1%) patients with complete hearing loss recovered. Other studies have also argued that severe hearing loss has a great correlation with poor outcomes.^12^, ^13^, ^14^, ^15^ Researchers indicated that initial hearing level above 72.5 dB HL contributed to poor prognosis.^14^ A complete recovery rate of 0.5% to 2.13% was observed in SSNHL patients with total hearing loss,^13^^,^^15^ which is consistent with our study.

It has already been demonstrated that the audiogram shape is a promising predictor of hearing recovery.^16^, ^17^, ^18^ In our study, the SHAP values of the ascending audiogram are greater than 0, which has a significant positive impact on prognosis. In contrast, the SHAP values of the profound shape are less than 0, indicating the largest negative contribution and the worst prognosis. Scholars has also found the ascending type has the best prognosis: 63%–88%,^17^ while only 14.29% of patients with profound type recovered.^10^ It can be concluded that it is essential to inform patients with profound type about the prognosis risk and focus on comprehensive management, such as hyperbaric oxygen therapy. Researchers suspected that the basal region of the cochlea which receptive to high frequencies is more prone to permanent damage than the upper regions, possibly because of differences in blood supply and metabolic needs.^18^

The recovery group had a much younger population than the non-recovery group. We detected the threshold for achieving favorable outcomes is 50.5 years. Advanced age has been consistently linked to decreased rates of hearing recovery and lower absolute threshold gains. Shimanuki et al.^14^ reported age ≥60-years declared a poor hearing outcome. Researchers found that the recovery rates decreased as age increased, the 18‒30-years, 31‒40-years, and 51‒60-years showed a recovery rate of 85%, 30%, and 32%, respectively.^15^ Age-related degeneration, specifically the Blood Labyrinth Barrier (BLB) has a higher vascular permeability in older patients, might explain the poor prognosis in older patients with SSNHL.^19^

Early treatment has consistently been emphasized by many previous studies.^3^ Our study illustrated delayed interval time suggested negative hearing outcomes. Researchers found the interval cutoff was 7, 10 and 14-days.^12^^,^^13^^,^^20^ However, no association was observed between hearing gain and time interval in patients with profound SSNHL.^13^ Hence, the poor initial hearing level itself is a more influential prognostic factor than the time interval, which is consistent with our finding.

Our study revealed that the non-recovery group has a significantly lower ALB level, with a cutoff value of 44.75. ALB level was also found to be a valuable prognostic factor for SSNHL in several studies. Zheng et al.^21^ discovered that the effective group had a markedly elevated level of ALB and Albumin to Globulin Ratio (AGR) compared to the non-effective group. And ALB level presented strong associations with the occurrence of SSNHL (OR = 0.809). Other studies displayed a statistically significant difference in mean CAR between SSNHL patients and the control group. However, no significant difference in recovery was detected.^22^^,^^23^ Meanwhile, the Fibrinogen to Albumin Ratio (FAR) levels were significantly greater in patients without recovery compared to both the recovery and the controls.^23^ One study found SSNHL patient complicated by hypertension had higher CAR levels than the non-hypertensive group, and an increase in CAR level also indicated a poorer prognosis.^24^ ALB has emerged as a strong marker of prognosis in individuals suffering from cardiovascular disease and stroke.^25^ This further implied a pathogenesis of vascular involvement in SSNHL. Since ALB is crucial for fluid exchange across the capillary membrane and maintaining endothelial permeability. Herein, the prognosis of SSNHL may be closely linked to decreased ALB concentration, which may be due to impaired labyrinthine perfusion caused by endothelial dysfunction.

MCV was found to be a predictive factor with a cutoff value of 88.85 for the first time. A previous study exhibited that mean Red blood cell Distribution Width(RDW) was significantly higher in the unrecovered group (13.2%) compared with the recovered group (12.7%) in patients with SSNHL, and RDW was significantly associated with hearing recovery (OR = 2.33, p = 0.012).^26^ RDW showed no significant difference between the two groups in our study. Nevertheless, RDW is an indicator of variation in Red Blood Cell (RBC) size, which is closely connected with MCV. Studies have confirmed MCV in relation to ischemic vascular diseases.^27^^,^^28^ MCV and RDW were also used to predict left atrial stasis in patients with non-valvular atrial fibrillation.^27^ Furthermore, a longitudinal study observed a higher MCV levels correlated with an increased risk of cerebrovascular or cardiovascular disease-related death.^28^ Increased MCV may have a significant impact on SSNHL as vascular compromise is one of the most widely accepted theories of SSNHL pathophysiology. The increase in blood viscosity caused by enlarged RBCs results in a significant reduction in blood flow in microvascular. The blood supply to the cochlea is further reduced, leading to a reduction in intracochlear oxygen levels. Irreversible injury could occur due to the extremely susceptible nature of cochlear structures to even brief episodes of hypoxia.

Several studies input large numbers of variables in model construction.^2^, ^3^, ^4^, ^5^, ^6^ Park et al.^6^ investigated the effectiveness of five ML models in 227 SSNHL patients using 32 variables. Uhm et al.^4^ analyzed the data of 244 SSNHL patients, 35 variables were used, and another model with 52 variables in 298 patients was assessed.^5^ Despite their fair predictive ability, these models cannot be popularized clinically due to the complexity of variables. Current study only included six easily available features, thus further increasing their general applicability.

The best predictive model was also obtained with RF according to our study. Similar result of RF model was also found (AUC of 0.74).^4^ The ability of RF to handle high-dimensional data and large-scale features makes it suitable for classification and regression tasks, such as our data. Bing et al.^2^ showed that the predictive power of the LR model was high when only three variables were analyzed (AUC = 0.80). However, as the number of variables increased, the predictive power decreased. LR model sustained a promising performance in our study. Hence, it remains a remarkable choice in handling concise variables.

We noticed that previous studies neglected to calibrate their results. Calibration is a vital component of model performance evaluation. It can be a useful tool in determining whether making decisions based on the model is harmful or not.^29^ Models in our study were well calibrated. Additionally, data from two different medical centers was incorporated into our study. Herein, our results have a high level of credibility and generalizability.

The limitations of this study require consideration. Firstly, although our study has a large sample size, it remains insufficient. ML models perform better as the sample size and variables increase, further study with an even larger sample size is greatly essential. Secondly, our results were not further validated in an external cohort. A more diverse population from multiple institutions as well as an external validation is needed to further validate the ML model. In addition, the MICE technique used for missing data may result in bias. Even so, the technique is still a recommended reference that is frequently used in data processing.

## Conclusion

The SHAP analysis showed that the degree of hearing loss, audiogram shape, duration between onset to treatment, age, MCV, and ALB levels are important features to predict hearing recovery. The models depending exclusively on a few convenient and easily available variables can be used as decision-making tools in daily practice.

## ORCID ID

Yaxuan Liu: 0009-0008-8838-2483

Feng Zhao: 0009-0005-1688-2444

Huiying Chen: 0000-0001-8751-0465

## CRediT authorship contribution statement

Jueting Wu: Conceptualization; Methodology; Software. Ruru Chen: Data curation; Investigation. Yaxuan Liu: Visualization; Writing-Original draft preparation. Feng Zhao: Supervision. Huiying Chen and Xiaoyu Lin: Software; Validation. Jiping Su: Writing-reviewing and Editing.

## Ethics approval and consent to participate

This retrospective study was performed in line with the principles of the Declaration of Helsinki, and approved by the Ethics Committee of Lishui People’s Hospital (nº 2024-056), the Second Affiliated Hospital and Yuying Children’s Hospital of Wenzhou Medical University (nº 2024-K-130-01).

## Funding

This research was funded by National Natural Science Foundation of China (nº 82260221) and Guangxi Natural Science Foundation (nº 2023GXNSFAA026288).

## Declaration of competing interest

The authors declare no have conflicts of interest.
